# Repeat posterior fossa exploration and external neurolysis for recurrent trigeminal neuralgia following microvascular decompression

**DOI:** 10.3389/fsurg.2025.1694389

**Published:** 2026-01-12

**Authors:** Zhong Liu, Jiao Xu, Ming-Liang Ren, Min-Hui Xu, Sha Chen, Xu-Zhi He, Xu-Hui Wang

**Affiliations:** 1 Department of Neurosurgery, Daping Hospital, Army Medical University, Chongqing, China; 2 Key Laboratory of Biochemistry and Molecular Pharmacology, Department of Pharmacology, Chongqing Medical University, Chongqing, China

**Keywords:** microvascular decompression, neurolysis, recurrence, Teflon, trigeminal neuralgia

## Abstract

**Objective:**

Recurrence of trigeminal neuralgia (TN) after microvascular decompression (MVD) poses a challenge for neurosurgeons. This study aimed to investigate the effect of repeat posterior fossa exploration (RPFE) and external neurolysis for TN recurrence following MVD.

**Methods:**

Totally, 38 TN patients who experienced recurrence after MVD were included. All patients underwent RPFE and limited neurolysis. The Barrow Neurological Institute Pain Intensity Scale were utilized to evaluate preoperative pain and postoperative outcomes.

**Results:**

The median follow-up period was 63.8 months (range: 5–112 months). Thirty-three out of the 38 patients achieved excellent outcomes, 4 had good outcomes, and 1 experienced failure. The rates of complete pain relief was 92% immediately post-surgery and 86.8% at the final follow-up. Re-exploration revealed Teflon adhesion in almost all patients (92%). Complications included new facial numbness (*n* = 5), temporary facial weakness (*n* = 3), hearing loss and tinnitus (*n* = 1), wound infection (*n* = 1), and postoperative hemorrhage (*n* = 1).

**Conclusions:**

Teflon adhesion was frequently observed upon re-exploration. A substantial majority of TN patients experiencing recurrence post-MVD achieved positive outcomes via RPFE and neurolysis. Complications, particularly facial numbness attributed to premature excessive Teflon dislodgement, continue to pose challenges. However, limited neurolysis, have proven effective in minimizing these complications.

## Introduction

Trigeminal neuralgia (TN) is recognized as one of the most severe pain syndromes, marked by recurrent, unilateral, paroxysmal, transient, and intense electric shock like pian in the trigeminal nerve distribution area. Neurovascular conflict (NVC) in the posterior fossa is identified as the primary cause of TN (1–3). Microvascular decompression (MVD) is the preferred treatment for patients with medication-refractory TN (4, 5). However, the long-term recurrence rate post-MVD is reported to be between 20% and 30% (1, 6, 7). Recurrence factors may include dislocation of the Teflon implant, excessive Teflon insertion, new vascular compression, and Teflon granuloma, acting either singly or in combination. In some cases, no definitive cause for recurrence is identified (8–10). Notably, intraoperative observations have more commonly identified Teflon adhesion—rather than prosthesis dislocation or new vascular compression—during re-exploration (11, 12). Consequently, Lysis of adhesions around the trigeminal nerve is deemed necessary in subsequent surgeries. Although neurolysis is considered both safe and effective, it is not devoid of complications, such as facial numbness. Thus, this study aims to identify more effective interventions to reduce the recurrence after the 1st MVD and minimize complications from a 2nd surgery by analyzing videos of both procedures.

## Methods

### Patients

Our study involved a retrospective analysis of 38 patients who experienced recurrent pain after MVD between 2014 and 2023 at our hospital, during which time over 1,500 patients underwent their initial MVD in our department. Of these, 35 patients received their 1st MVD with us, while 3 were initially treated at other institutions and later underwent repeat surgery at our center. The inclusion criteria included a history of TN, a definite period of being pain-free following the initial MVD, failure to respond to medication or inability to tolerate its side effects, and recurrence of facial pain on the same side. Idiopathic TN was diagnosed following the criteria of the International Classification of Headache Disorders, 3rd edition (ICHD-3). Data were collected and analyzed retrospectively from all patients. Although an MRI scan was conducted before the 2nd surgery, including 3D T1– and T2-weighted high-resolution sequences, the presence of vascular compression could not be definitively confirmed due to the challenge of identifying these subtle structures amidst Teflon imaging around the nerves.

This study received approval from the Ethics Committee of Army Medical Center of the PLA.

### Preoperative management

A preoperative MRI examination was performed in all patients, including 3D– and T2 weighted high-resolution sequences, for clear visualization of the trigeminal nerve and all vascular structures. The use of 3D time-of-flight Magnetic Resonance Angiography (MRA) allowed visualization of only vessels with high flow, which are principally arteries. However, the vascular statistics in the study were based on intraoperative observations.

### Surgical techniques employed in the second surgeries

**Figure 1:**
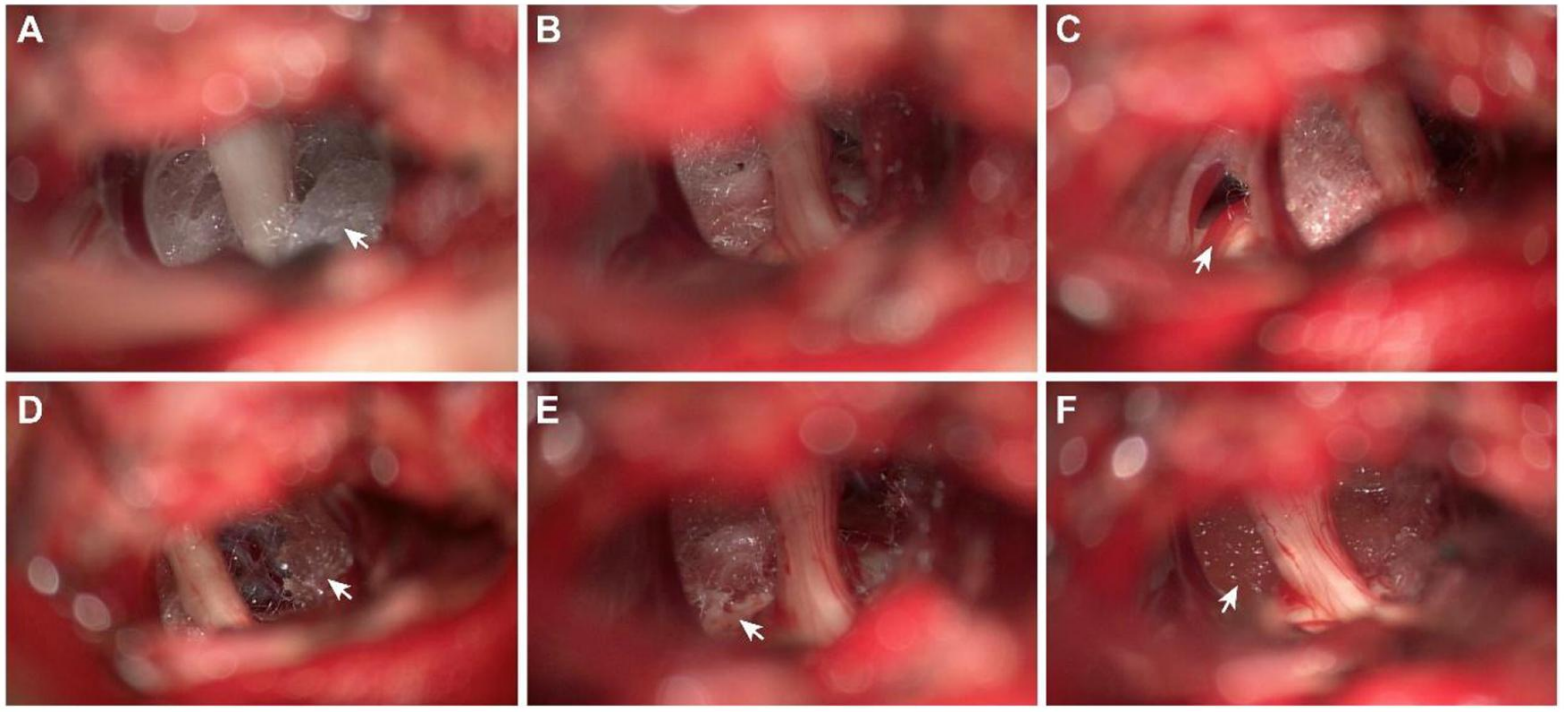
**(A–F)** depicts the intraoperative observations and interventions in patients with recurrent TN. **(A)** Teflon encased the trigeminal nerve without visible adjoining vessels. **(B)** A portion of the Teflon was excised using scissors, revealing no vessels traversing the nerve. **(C)** The SCA (the previous offending vessel), identified as the previously offending vessel, was fixed to the brainstem, distanced from the trigeminal nerve by Teflon adhesions. **(D)** Teflon was carefully separated from the arachnoid and the nerve's perilemma, leaving a significant amount of Teflon medial to cranial nerves VII and VIII. **(E)** Following neurolysis, the trigeminal nerve was free of any Teflon contact. However, the dissection resulted in some damage to the leptomeninges on the brainstem. **F**: Post-neurolysis, gelatin was applied to segregate the trigeminal nerve from the remaining Teflon.

Following the induction of general anesthesia, patients were positioned laterally with three-point fixation. The surgeries utilized intraoperative monitoring of brainstem auditory-evoked responses and were conducted through an enlarged retrosigmoid craniotomy approach. Upon opening the dura, any adhesions between the cerebellum and dura were meticulously dissected, and the area surrounding the trigeminal nerve was carefully examined. Initially, Teflon was removed as comprehensively as possible, followed by vascular decompression using new materials (Complete decompression) (13). In cases where no apparent vessels were observed near the trigeminal nerve, either partial nerve section (PNS) or nerve combing was undertaken after Teflon removal. Generally, for PNS, the inferior one third to one half of the portio major was sectioned (14). For nerve combing, the trigeminal nerve was longitudinally divided along its fibers using a special nerve combing knife with a cutting edge of 0.90 mm into 3–5 bundles from the nerve root entry zone (REZ) to the petrous bone (9). Starting in 2016, we adopted a method of limited neurolysis similar to that described by Bakker et al. (9, 15), including: 1) Sharp dissection of arachnoid adhesions around the trigeminal nerve; 2) Removal of partial Teflon between the nerve and tentorium, skull base or brainstem, break their connection, and keep the nerves in a relaxed state. 3) Avoiding stripping Teflon too close to the nerve to prevent nerve injury. If offending vessels were immobilized by adhesion or scarring, gelatin sponges were inserted between the nerves and any remaining Teflon (12), no new materials were introduced (Figures 1A–F, Supplementary Video 1). All procedures were led by the senior author (XHW).

### Assessment of pain and follow-up

Recurrent TN was identified as the return of TN pain on the same side following a MVD procedure that initially achieved complete relief without the need for medication. The Barrow Neurological Institute (BNI) Pain Intensity Scale scores were employed to evaluate preoperative pain and postoperative outcomes, assessed by an independent neurosurgeon not involved in patient care (16). Postoperative outcomes were categorized as “complete pain relief” (BNI pain score: I), “partial pain relief” (BNI pain score: II–III), or “failure” (BNI pain score: IV–V). These outcomes were evaluated immediately post-surgery and at the final follow-up visit, either in the outpatient department or via telephone. Patients were inquired about their facial sensation, the intensity of any residual pain episodes, and any medications taken for TN.

### Statistical analysis

SPSS statistical software version 24.0 (IBM Corp, Armonk, NY, USA) was utilized for data analysis. Means were reported for quantitative data. Descriptive statistics summarized clinical features and patient characteristics. The chi-square test, Fisher's exact test, and Spearman correlation test were employed for statistical analysis. Values of *P* < 0.05 were deemed statistically significant. The Kaplan–Meier product limit method determined the duration for which patients maintained excellent facial outcomes after their 1st surgery**.**

## Results

### Patient characteristics

The study group comprised 20 men and 18 women, with a median age of 65 years old (range: 40–82 years), who underwent their first MVD between 2009 and 2023 (Table 1). In contrast, a significant number of patients with recurrent conditions opted for noninvasive treatments or continued with oral medications. The median duration of pain among these patients after the first MVD procedure was 7.39 years (rang: 0.5–30 years). The majority had symptoms on the right side (20/38, 53%), and the maxillary and/or mandibular divisions were affected in most cases (35/38, 92%). All patients experienced a pain-free period exceeding one month following their initial MVD (range: 1–96 months; median: 22.64 months), and the median interval between the first and subsequent surgeries was over 36 months (range: 3–100 months). All patients had typical TN pain before both the first and subsequent surgeries and suffered from medically intractable classical TN pain attacks (BNI V). The majority were responsive to carbamazepine or oxcarbazepine prior to the 1st and 2nd surgeries (90% and 80%, respectively), though the side effects were intolerable. Ten patients had undergone neurorestorative procedures, including glycerol injection, thermocoagulation, and Gamma knife surgery, since the onset of TN, five of them experienced facial numbness afterwards.

**Table 1:** Demographic and clinical characteristics of patients with TN undergoing repeat MVD.

Characteristics	*N* (%)
Cases	38
Male sex	20 (53%)
Age	
	65
	40–82
Affected side	
Left	18
Right	20
Typical symptoms	
Before 1st MVD	38 (100%)
After recurrence	38 (100%)
Pain location	
V1	1
V2	11
V3	10
V1 + V2	2
V2 + V3	13
V1 + V2 + V3	1
Duration of pain (yrs)	
Median	7.39
Range	0.5–30
Interval between operations (mos)	
Median	36.80
Range	3–100
Time to recurrence after 1st op (mos)	
Median	22.64
Range	1–96
Follow up period (mos)	
Median	63.8
Range	5–112
Previous ablative procedures	
None	28
Glycerol injection	6
Thermocoagulation	2
Gamma knife	7

### Operative findings

**Figure 2:**
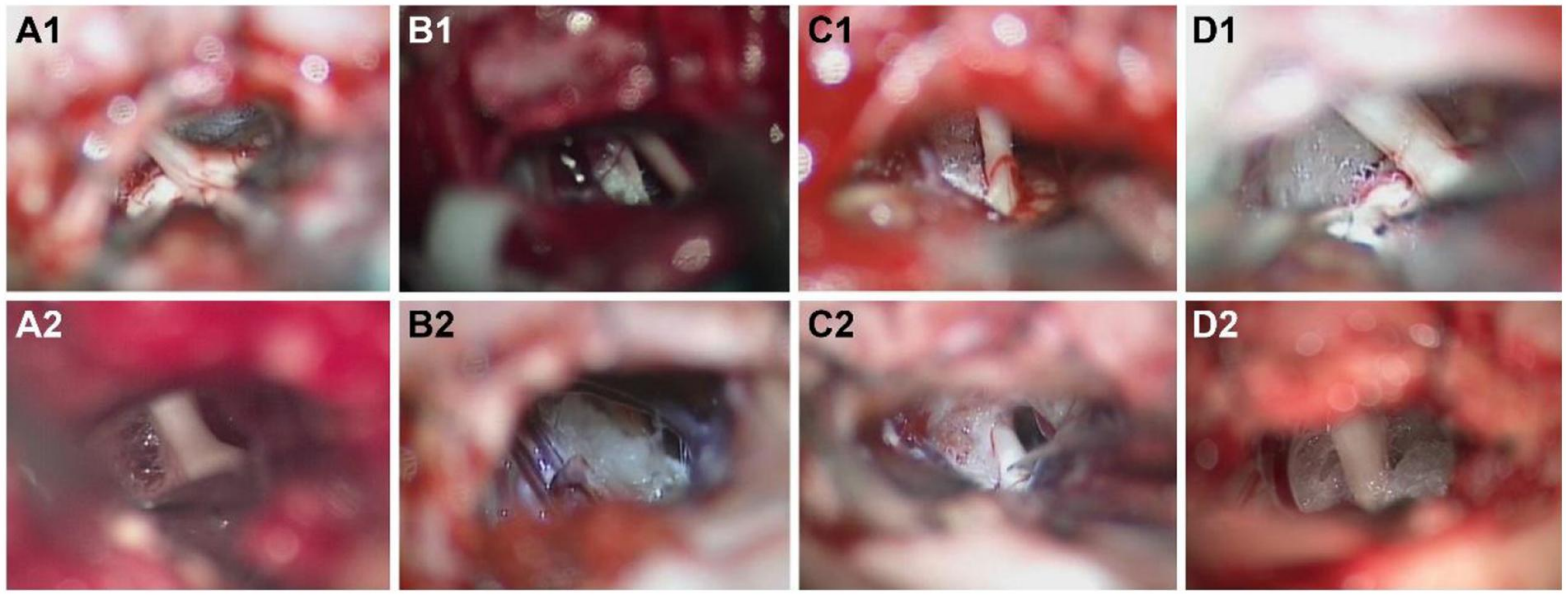
**(A–D)** depict imaging from 4 patients with recurrent TN. **A1-D1** illustrate the status of trigeminal nerve decompression following the completion of the 1st MVD, highlighting the adequate space surrounding the nerves. (**A2–D2**) display the condition of the nerve prior to the commencement of the 2nd neurolysis surgery. Here, the Teflon has expanded, encroaching upon the space around the trigeminal nerve and adhering to the surrounding tissues.

During the second surgery, Teflon adhesion was observed in 35 cases (Figure 2), arachnoid scar tissue in 32 cases, arterial compression in 2 cases, venous contact in 1 cases and Teflon granuloma in 1 case. No new offending vessels or displacement of Teflon were detected (Table 2). However, either Teflon adhesion or arachnoid scar tissue, or both, were present in all cases. Among the 35 cases with Teflon adhesion, sharp dissection and limited neurolysis were carried out, then a gelatin sponge was positioned between the nerve and the existing Teflon remnants. In 3 cases of vessel compression, Observation revealed that the vessels implicated in the 1st MVD had become adhered in place (Figure 1C). Consequently, rather than applying new Teflon, a gelatin sponge was positioned between the nerve and the existing Teflon remnants, following the approach documented by Li et al. (12).

**Table 2:** Intraoperative findings in 38 patients with TN undergoing repeat MVD.

Intraoperative findings (*n* = 38)	Number (%)
Teflon adhesion	35 (92.1%)
Arachnoid scar tissue	32 (84.2%)
Arterial compression	2 (5.3%)
Venous contact	1 (2.6%)
Teflon granuloma	1 (2.6%)
Dislocation of Teflon	0 (0%)

### Outcomes and complications

Among the 38 patients, 34 (89.5%) experienced complete pain relief immediately following the second surgery, while the remaining 4 patients saw a reduction in facial pain within 10 days, as reported by Deng et al. (17). One patient passed away at the last follow-up without experiencing a recurrence of pain postoperatively. During a follow-up period ranging from 5 to 112 months (median, 63.8 months), 33 patients reported excellent (BNI pain score: I, 33/38) or good (BNI pain score: II–III, 3/38) outcomes (Table 3). Facial numbness emerged as the most common complication, with 5 patients developing new facial numbness after aggressive neurolysis (Table 4). Consequently, we increasingly recognize that limited neurolysis, as described by Bakker et al. (15), is a safer approach that may help mitigate the risk of facial numbness post-surgery. Additionally, 3 patients experienced temporary facial weakness, which resolved within 2 weeks. Other postoperative complications included hearing loss and tinnitus (1 patient), temporary ataxia (1 patient), and infection (1 patient). The most severe complication encountered was hemorrhage, from which the patient ultimately recovered well.

**Table 3:** Surgical outcomes for 38 patients with TN undergoing repeat MVD.

BNI pain intensity score	Immediate postoperative	Last follow-up
I	34	33
II	3	1
III	1	2
IV	0	1
V	0	1

**Table 4:** Complications from repeat surgery for persistent recurrent TN.

Complication	Cases
Hemorrhage	1
CSF leakage	0
Facial numbness (new)	5
Hearing loss and tinnitus	1
Temporal facial weakness	3
Wound Infection	1

## Discussion

Among the available treatments for refractory TN, MVD is favored as the primary option due to its proven efficacy and safety (18). However, only 64%–73% of patients remain pain-free without medication ten years after undergoing a single MVD procedure (1, 19, 20). Factors contributing to relapse include incomplete decompression, Teflon felt displacement, new vascular compression, and notably, Teflon adhesion and granuloma formation, with recent evidence increasingly highlighting Teflon adhesion as a significant risk factor for TN recurrence (8, 11, 12, 16, 21). In this study, Teflon adhesion was observed in all examined cases. Our imagery corroborates findings by Amador et al. (21) and Chen et al. (22), showing Teflon expansion encroaching upon the trigeminal nerve space. Such adhesions increase nerve tension and reduce compliance, akin to a “piston-effect” where artery pulsations from systole to diastole are transmitted to the trigeminal nerve through the interposed and hardened Teflon (9, 10). Consequently, we propose that diminished trigeminal nerve compliance may underlie TN recurrence in these instances. Notably, novice surgeons, eager to prevent vessel rebound, might overuse Teflon to ensure separation from the trigeminal nerves, potentially leading to a heightened recurrence rate.

Comparative analysis of photographs and videos from the 1st and 2nd surgeries revealed that Teflon tends to expand, eventually encroaching upon the space surrounding the trigeminal nerve (21), and adheres to nearby structures, including the tentorium, vessels, brainstem leptomeninges, arachnoid, and trigeminal nerve peri lemma (Figure 2). Teflon was found to firmly adhere to the tentorium and vessels, making dissection particularly challenging. In contrast, Teflon's adhesion to the arachnoid and trigeminal nerve peri lemma was looser, facilitating easier dissection. Nonetheless, separating Teflon from the brainstem proved to be only partially achievable due to the presence of small vessels on the brainstem's surface, which tended to tightly bind to the Teflon. Therefore, sharp dissection is recommended as a safer and more efficient method in such cases. Interestingly, direct compression of the trigeminal nerve by Teflon was not observed in any patient. Instead, Teflon anchored the trigeminal nerve to the tentorium, petrous dura, or brainstem through adhesions, thereby increasing nerve tension, complicating mobility (Figure 1C).

As highlighted, Teflon adhesion emerges as a significant risk factor for the recurrence of pain. Consequently, it is advisable to minimize the use of Teflon and adopt surgical techniques that prevent its adhesion during MVD. Teflon placement should be separation of the neurovascular confliction rather than isolation between the nerve and the vessel (23, 25). It is essential to prevent any contact between the prostheses and the trigeminal nerve throughout its entire course. This method has been shown to potentially lower the recurrence rate post-MVD (24). According to Li, the purpose of Teflon is to displace the offending vessels from the trigeminal nerve and ensure this separation persists (25). Over time, these Teflon placements can facilitate the adhesion of the vessel to either the brainstem or the dura, thus preventing a return to the previous state, a scenario often encountered in subsequent surgeries. In situations where the arterial loop is excessively elongated or the space around the trigeminal nerve is restricted, the suspension of the offending vessel using a stitch or glue is recommended (11).

In recent years, numerous studies have demonstrated that non-contact methods such as the “sling” or “clip” techniques, or securing the offending artery away from the TN using adhesive (26, 27), can yield favorable outcomes. These approaches help minimize the use of Teflon felt and reduce the likelihood of postoperative complications, thereby decreasing the recurrence rate after surgery. However, the complexity of these surgical techniques can lead to severe complications. For instance, Jiang Liu et al. reported 14 cases of postoperative hemorrhage occurring on average 41.75 h after employing the biomedical glue sling technique to reposition the VBA towards the petrosal apex during microvascular decompression (28). Consequently, further research is imperative to ascertain the safety of these technologies, especially in cases involving complex vertebral arteries impinging on the trigeminal nerve. Therefore, it is essential to tailor treatment to individual patients during surgery, taking into account the type of offending vessel, the dimensions of the posterior fossa space, and the surgeon's proficiency with the technique.

Our result align with those reported in recent studies (3, 12, 29, 30), where all patients exhibited positive outcomes during the follow-up period (median, 39.5 months). The patient selection process likely played a role in achieving these favorable outcomes. In our cohort, recurrent facial pain was characterized as typical TN pain, with the majority of patients (36/38) responding variably to carbamazepine or oxcarbazepine. These factors collectively suggested a favorable postoperative prognosis (7, 17). The profile of postoperative complications mirrored that seen in similar studies, with facial numbness being the most prevalent issue. This was not attributed to damage to trigeminal nerve structures themselves but possibly to injury to vessels on the surface of the trigeminal nerve. During the second neurolysis procedure, we successfully decreased the incidence of facial numbness by meticulously detaching the Teflon from the trigeminal nerve.

In the initial phase, Teflon removal was attempted as comprehensively as possible to decompress the nerve (complete decompression), as outlined by previous studies (13). However, this proved challenging due to the Teflon's tendency to adhere strongly to the tentorium and adjacent vessels. In a notable case, efforts to extract Teflon proximal to the trigeminal nerve resulted in leptomeningeal damage to both the brainstem and trigeminal nerve surfaces. Such injuries may lead to postoperative hemorrhage and a cascade of severe complications. Additionally, the inadvertent harm to the small vessels nourishing the trigeminal nerve during this process could precipitate postoperative facial numbness. Consequently, we adopted a strategy of limited neurolysis detailed earlier (Figures 1A–F) to sever the Teflon's bonds to nearby structures through targeted neurolysis, as recommended by Bakker et al. (15). This method proved effective, notably reducing the incidence of facial numbness. Meanwhile, to avoid potential complications, alternative approaches—such as the Subtemporal Transtentorial Approach or the subtemporal intertentorial—should be considered. This technique allows exposure of the entire cisternal segment of the trigeminal nerve (including the REZ) through a tentorial incision, facilitating more effective and thorough neurolysis. The wider and shallower surgical field also provides better visualization, making it easier to protect adjacent nerves and blood vessels (31, 32).

It should be noted, however, that offending vessels and the trochlear nerve may adhere to the tentorium due to Teflon implantation or scar formation. Therefore, when incising the tentorium, meticulous care is required to avoid injury to these structures.

This study possesses several limitations. Firstly, it is retrospective in nature. Secondly, preoperative MRI scans do not provide a definitive correlation between Teflon placement and TN. Thirdly, the pressure exerted on the trigeminal nerve cannot be quantified during surgery, precluding any comparative analysis of pressure changes. These limitations are not unique to this study but are present in another research as well. Future studies should aim to enhance both the accuracy and efficacy of treatments for trigeminal neuralgia.

## Conclusions

In this cohort, the majority of patients experienced either excellent or good outcomes. To minimize the risk of recurrence following an initial MVD, Teflon should be employed to secure the offending vessels after comprehensive dissection; however, it should not be placed between the trigeminal nerve and the offending vessels. For secondary surgeries, limited neurolysis and precise dissection is advised to mitigate the risk of facial numbness and significant complications. Overall, repeat surgeries have been demonstrated to be safe; with the exception of a single case in our study, no severe complications were reported. Consequently, for patients experiencing recurrence of TN, revision surgery should be more strongly advocated.

## Data Availability

The original contributions presented in the study are included in the article/Supplementary Material, further inquiries can be directed to the corresponding authors.
